# The impact on neonatal mortality of shifting childbirth services among levels of hospitals: Taiwan's experience

**DOI:** 10.1186/1472-6963-9-94

**Published:** 2009-06-08

**Authors:** Shi-Yi Wang, Sylvia H Hsu, Li-Kuei Chen

**Affiliations:** 1 Health Policy and Management, School of Public Health, University of Minnesota, 420 Delaware Street, S.E., Minneapolis, MN, 55414, USA; 2 Ton-Yen General Hospital, 69 XianZheng 2nd Street, Chu-Pei City, Hsin-Chu County, Taiwan; 3 Schulich School of Business, York University, 4700 Keele Street, Toronto, ON M3J 1P3, Canada; 4 Department of Anesthesiology, National Taiwan University Hospital, No.7, Chung-Shan S. Road, Taipei, 100, Taiwan

## Abstract

**Background:**

There is considerable discussion surrounding whether advanced hospitals provide better childbirth care than local community hospitals. This study examines the effect of shifting childbirth services from advanced hospitals (i.e., medical centers and regional hospitals) to local community hospitals (i.e., clinics and district hospitals). The sample population was tracked over a seven-year period, which includes the four months of the 2003 severe acute respiratory syndrome (SARS) epidemic in Taiwan. During the SARS epidemic, pregnant women avoided using maternity services in advanced hospitals. Concerns have been raised about maintaining the quality of maternity care with increased demands on childbirth services in local community hospitals. In this study, we analyzed the impact of shifting maternity services among hospitals of different levels on neonatal mortality and maternal deaths.

**Methods:**

A population-based study was conducted using data from Taiwan's National Health Insurance annual statistics of monthly county neonatal morality rates. Based on a pre-SARS sample from January 1998 to December 2002, we estimated a linear regression model which included "trend," a continuous variable representing the effect of yearly changes, and two binary variables, "month" and "county," controlling for seasonal and county-specific effects. With the estimated coefficients, we obtained predicted neonatal mortality rates for each county-month. We compared the differences between observed mortality rates of the SARS period and predicted rates to examine whether the shifting in maternity services during the SARS epidemic significantly affected neonatal mortality rates.

**Results:**

With an analysis of a total of 1,848 observations between 1998 and 2004, an insignificantly negative mean of standardized predicted errors during the SARS period was found. The result of a sub-sample containing areas with advanced hospitals showed a significant negative mean of standardized predicted errors during the SARS period. These findings indicate that despite increased use of local community hospitals, neonatal mortality during the SARS epidemic did not increase, and even decreased in areas with advanced hospitals.

**Conclusion:**

An increased use of maternity services in local community hospitals occurred during the SARS epidemic in Taiwan. However, we observed no increase in neonatal and maternity mortality associated with these increased demands on local community hospitals.

## Background

Regionalization of perinatal care, which links a tiered structure of facilities and refers women with high-risk pregnancies to a central facility with advanced technology and increased staff, has been established to improve perinatal health care and decrease neonatal mortality [1-3]. Studies have demonstrated that the relative risk for low birthweight infants in local community hospitals is significantly higher than that in advanced hospitals, ranging from 1.3 to 2.3 [4-6]. Although the benefits of perinatal care for low birthweight infants in advanced hospitals are well established, the data on the outcome of infants of normal birthweight are still inconclusive [7-15]. The public considers advanced hospitals, with their sophisticated technology and equipment, safe places for both high- and low-risk deliveries because undetectable prenatal conditions can cause unexpected complications during childbirth. Even though several studies on low-risk pregnancy show no statistically significant difference in neonatal mortality rates between low technology facilities and advanced technology hospitals [7-10], there is still evidence of increased risks for low-risk deliveries in local community hospitals [11-15]. For example, Heller et al. reported a more than three-fold risk of neonatal death in small hospitals compared to large hospitals [15]. These inconsistent results have raised concerns about the impact of regionalization on the outcome of low-risk deliveries.

In Taiwan, the accreditation system classifies medical institutions into four categories: medical centers, regional hospitals, district hospitals and clinics. Medical centers and regional hospitals provide neonatal intensive care for high-risk pregnancies, while district hospitals provide premature observation care for mild-risk pregnancies. Ob-gyn clinics are run by obstetrics-gynecology specialists and provide medical care for women, including low-risk child deliveries. In Taiwan, low-risk pregnant women are allowed to seek services from medical centers without restrictions. During the 2003 SARS epidemic in Taiwan, the general population avoided seeking health care due to a combination of factors, including the vulnerability of health-care workers, and the rapid transmission of and limited knowledge about the disease [16]. In particular, people avoided seeking care from advanced hospitals (i.e., regional hospitals and medical centers) because SARS patients were being treated there. Therefore, expectant mothers began seeking maternity services at local community hospitals instead of at advanced hospitals to avoid becoming exposed to SARS [17]. This change in expectant mothers' preference of healthcare providers led to an increase of 7.1 % and 2.1% of the market share of total childbirth deliveries in clinics and in district hospitals, respectively [17]. Due to inconclusive evidence surrounding birth outcomes in local community hospitals, this large shift in childbirth services to local community hospitals has led to serious concerns about quality of care [17]. Therefore, this study undertook a population-based examination of neonatal and maternal mortality between 1998 and 2004 to investigate the impact of an increase in deliveries in district hospitals and clinics during the 2003 SARS epidemic.

## Methods

The analysis was based on data from Taiwan's National Health Insurance annual statistics, which included detailed monthly and county maternal and neonatal mortality numbers. We retrieved the data from 1998 to 2004 to compare the impact of the shift in childbirth services during the SARS epidemic, which took place from May 2003 to August 2003 [17]. Examining the data from the period after the SARS epidemic (i.e., post September 2003) allowed us to rule out the effect of technological progress on the outcome of maternity services, which might mitigate the potential negative impact of the shift in treatment from May to August 2003. We applied an interrupted time-series design to analyze the effect of shifting childbirth services from one hospital level to another.

Both neonatal and maternal mortalities were analyzed to examine the impact of shifting hospital services on childbirth outcomes. For each county, monthly neonatal and maternal mortality rates were calculated; neonatal rates were determined by dividing the number of neonatal deaths by the number of childbirths, and maternal mortality rates were determined by dividing the number of maternity deaths by the number of childbirths. Because the small number of maternal mortality cases precludes a meaningful analysis, descriptive statistics are presented.

Linear regressions were estimated using data from the years 1998 to 2002 to examine the changes in neonatal mortality rates for the pre-SARS period. A total of 22 counties were included in the analysis. We excluded the data from three isolated islands with no advanced medical institutions and small populations because the expense of transportation in these locations may have prevented expectant mothers from voluntarily selecting advanced hospitals and little shifting would have occurred. Furthermore, the SARS patients were found only on Taiwan's main island.

The dependent variables were monthly county neonatal mortality rates; the independent variables included "trend," a continuous variable that measured for the effect of yearly changes, and two binary variables, "month" and "county," which controlled for seasonal and county-specific effects. With the estimation results, we calculated the predicted mortality rate for each month and county from 1998 to 2004. The differences between observed mortality rates and predicted mortality rates were standardized with the standard error of individual predicted values.

The data were managed with SAS software, version 9.1.3. All analyses were tested for a significance level by using á value of 0.05. Because only secondary data are analyzed, no Institutional Review Board (IRB) approval is necessary.

## Results

**Figure 1:**
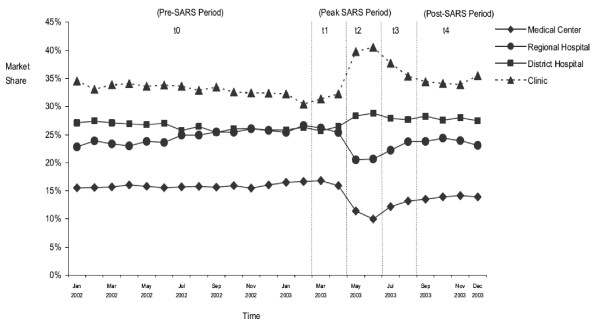
**Trends in market shares of childbirth services in Taiwan by provider's level, January 2002–December 2003**. Adapted from Lee et al. *BMC Public Health *2005, **5:**30–36.

Figure 1 is adapted from Lee et al. and shows the changes in childbirth services in hospitals of different levels [17]. Table 1 is adapted from *Health and Vital Statistics of Taiwan 2004 *and shows the number of neonatal and maternal deaths from 1998 to 2004. Neither the neonatal mortality rate nor the maternal mortality rate for the year 2003 was higher than for other sample years.

**Table 1:** Neonatal and maternal mortality of Taiwan Island, 1998–2004

	**Neonatal mortality**		**Maternal mortality**	
		
	Deaths	Rate (‰)	Deaths	Rate (‰)
1998	918	3.38	24	0.09
1999	980	3.45	24	0.08
2000	1,038	3.40	24	0.08
2001	865	3.32	18	0.07
2002	745	3.01	19	0.08
2003	624	2.75	15	0.07
2004	623	2.88	12	0.06

Data are extracted from *Health and Vital Statistics of Taiwan 2004.*

To compare the mortality rates for the SARS period of May to August 2003 with the same months of other sample years, we aggregated the monthly mortality rates into a four-month period. After the exclusion of the data from the three isolated islands, the number of childbirths from 1998 to 2004 was 1,798,369 and the number of neonatal mortality cases was 5,747. Table 2 shows a decrease in neonatal mortality rates over time, which did not increase during the SARS epidemic.

**Table 2:** Descriptive data on neonatal mortality, categorized by four-month periods: January to April, May to August, September to December, 1998–2004

	**January to April**		**May to August**		**September to December**	
			
	Number of Deaths	Rate (‰)	Number of Deaths	Rate (‰)	Number of Deaths	Rate (‰)
1998	304	3.32	318	3.59	292	3.25
1999	319	3.48	305	3.25	349	3.65
2000	322	3.41	356	3.65	355	3.19
2001	267	3.04	302	3.59	287	3.32
2002	240	3.05	271	3.41	229	2.62
2003	203	2.74	201	2.83	214	2.67
2004	189	2.74	213	3.16	211	2.70

Using the analysis of a linear regression model based on pre-SARS observations, Table 3 summarizes the univariate statistics of predicted errors of monthly county mortality rates. We applied the standard error of an individual predicted value to obtain standardized predicted errors. The mean of standardized predicted errors of 22 counties during the SARS epidemic period (i.e., May–August 2003) is -0.10 (95% confidence interval: -0.34–0.14), which indicates that the predicted values are insignificantly different from the observed values during the SARS period. This result demonstrates that, despite an increased use of local community hospitals, neonatal mortality during the SARS epidemic was lower, even though the difference was insignificant.

**Table 3:** The univariate statistics of standardized predicted errors of monthly county mortality rates

**Panel A: Standardized predicted errors of monthly county mortality rates are based on the model based on the total sample during the years 1998–2004**			
	SARS period (May – August 2003)	Pre-SARS period (January 1998–April 2003)	Post-SARS period (September 2003–December 2004)
Standardized predicted errors (95% CI)	-0.10 (-0.34–0.14)	-0.00 (-0.06–0.04)	-0.00 (-0.10–0.08)
**Panel B: Standardized predicted errors of monthly county mortality rates are based on the model based on the sub-sample of areas with hospitals of more than 1,000 beds**			
Standardized predicted errors (95% CI)	**-0.42** **(-0.74– -0.11)**	0.01 (-0.06–0.08)	-0.06 (-0.19–0.06)

We formed a sub-sample containing 12 counties where there were hospitals with more than 1,000 beds. The impact of shifting services would be greater in these areas because switching from one hospital level to another tends to occur more often there. The sub-sample presents a significantly negative mean of standardized predicted errors, (-0.42, CI (-0.74 – -0.11)) during the SARS period. This evidence indicates that the neonatal mortality rate in areas with large hospitals was significantly lower than predicted, despite the shift of childbirth services to local community hospitals during the SARS epidemic.

With the aggregation of the county data from Year 1998 to Year 2002, we recalculated total neonatal mortality rates of the pooled data and estimated a linear regression model with "month" and "trend" variables. Table 4 presents the observed monthly mortality rates and predicted mortality rates of Year 2003, which provides the comparison of the predicted errors of four months before the SAS, of the SARS period and of four months after the SARS period. Similar to the results of monthly county mortality rates, the analysis of the aggregated data shows negative predicated errors during the SARS period of May 2003–August 2003. The aggregated data of counties with large hospitals demonstrates a significant predicted error in July 2003, which indicates that in counties with advanced hospitals of more than 1,000 beds, neonatal mortality was significantly reduced in July 2003, despite an increased use of childbirth services in local community hospitals.

**Table 4:** Observed monthly mortality rates and predicted mortality rates based on the aggregated county data

Panel A: The aggregated monthly mortality rates of 22 counties			
	Predicted mortality Rate (‰) (95% CI)	Observed mortality rate (‰)	Standardized predicted errors
January 2003	3.28 (2.11–4.45)	3.56	0.48
February 2003	3.04 (1.87–4.20)	2.86	-0.30
March 2003	3.01 (1.84–4.17)	2.09	-1.58
April 2003	2.79 (1.62–3.96)	2.45	-0.58
May 2003	3.39 (2.22–4.55)	2.97	-0.71
June 2003	3.54 (2.37–4.71)	3.45	-0.15
July 2003	3.23 (2.06–4.40)	2.24	-1.70
August 2003	2.86 (1.69–4.03)	2.70	-0.28
September 2003	3.28 (2.11–4.45)	2.60	-1.18
October 2003	2.97 (1.80–4.14)	2.98	0.01
November 2003	2.93 (1.76–4.10)	2.89	-0.07
December 2003	2.66 (1.49–3.83)	2.24	-0.72
			
Panel B: The aggregated mortality rate of 12 counties where contain hospitals with more than 1,000 beds			
January 2003	3.04 (1.86–4.23)	3.31	0.45
February 2003	2.85 (1.67–4.03)	3.12	0.46
March 2003	2.95 (1.77–4.14)	2.14	-1.39
April 2003	2.70 (1.52–3.88)	2.41	-0.48
May 2003	3.34 (2.16–4.53)	3.25	-0.16
June 2003	3.70 (2.52–4.89)	3.13	-0.98
July 2003	3.25 (2.06–4.43)	**1.82**	**-2.42**
August 2003	2.98 (1.80–4.16)	2.50	-0.81
September 2003	3.26 (2.07–4.44)	2.43	-1.41
October 2003	2.98 (1.79–4.16)	2.64	-0.58
November 2003	2.87 (1.69–4.06)	2.42	-0.77
December 2003	2.55 (1.37–3.73)	1.97	-0.98

## Discussion

Normal birthweight or low-risk deliveries account for the majority of childbirth experiences. Although regionalized perinatal care is well-established for high-risk deliveries, it is crucial to examine the outcomes of normal birthweight deliveries in local community hospitals. The issue of whether high-technology hospitals provide better quality of care for normal birthweight deliveries than small maternity units has been examined extensively; however, the literature shows conflicting results regarding the outcome of normal birthweight infants in local community hospitals [7-15]. Due to these inconsistent results, the concern about quality of care as a result of the shifting of maternity services from advanced hospitals to local community hospitals associated with the SARS epidemic is understandable [17]. This study has shown that neonatal mortality during the SARS period did not increase. Hence, this evidence resolves the questions that Lee et al. raised about the impact of SARS on the shifting of childbirth services between hospitals of different levels [17].

We assumed that the majority of the shifts in childbirth services during the SARS event involved low-risk deliveries. At present, the National Health Insurance (NHI) program of Taiwan provides ten free antenatal clinics, which help obstetricians in local community hospitals assess high-risk pregnancies that they are then required to refer to regional hospitals and medical centers. Furthermore, obstetricians in local community hospitals have also been referring these high-risk patients to medical centers more often, due to the legal concerns associated with the complications inherent in high-risk deliveries and the increased malpractice lawsuits in Taiwan. In addition, Lee et al.'s study of the impact of the SARS epidemic on childbirth shows a 2.2% increase in the cesarean section rate in medical centers, but no increase in the cesarean section rate in local community hospitals during the SARS period, which implies that the increased services provided by local community hospitals involved low-risk deliveries [17]. Therefore, our assumption that the majority of childbirth cases that shifted from high- to lower-level hospitals involved low-risk deliveries is reasonable.

Our results echo the results of similar studies that tracked the outcome of low-risk births [7-10], but contradict predictions of a worse outcome for low-risk deliveries in local community or small hospitals [11-15]. A possible explanation for our findings of the similar outcome in both advanced and local community hospitals is that the analysis is based on the data of the most recent sample period, which is characterized by improved monitoring at local community hospitals. Frequent monitoring and quick detection thanks to more advanced technologies, such as bedside monitoring machines, have been allowing obstetricians to take appropriate precautions and avoid complications. However, the results of studies concluding lower neonatal mortality rates in advanced or large hospitals than in local community hospitals sampled the birth data before 1999 [11-15]. For example, using German data from 1990 to 1999, Heller et al. found that birthweight-specific mortality rates were lowest in large delivery units and highest in smaller delivery units [15]. Similar findings were documented in the study with the Norwegian data from 1972 to 1995 [14] and data from the United States in 1980 [13].

Research on birth settings for women with low-risk pregnancies often involves methodological challenges, such as small samples, non-random samples, differences between women who choose local community versus technology-advanced hospitals, confounding factors associated with inconsistencies in physician behavior, and data limitations [18]. This study applied a population-based approach to research the change in neonatal mortality during the increased use of local community hospitals associated with the SARS epidemic. The exogenous nature of the SARS event mitigates the problem of confounding factors as they relate to characteristics of expectant mothers and physician behavior among levels of hospitals. Issues surrounding non-random sampling are also moot because we used data on neonatal mortality for the entire newborn population in Taiwan between 1998 and 2004. Furthermore, this study's large sample size of 1,848 observations allows us to demonstrate clearly that the shifting of childbirth services among hospitals associated with the SARS epidemic did not increase the risk of neonatal deaths. In contrast, we found that the neonatal mortality rate decreased in areas that contained large hospitals which were more likely to incur the shifting of childbirth services. We acknowledge a limitation of this study that we did not directly measure neonatal mortality among hospitals because of data availability. Considering the small effect size associated with the impact of a 9.2% service shifting among hospitals, we recognize potential weak statistical power of our sample in concluding the outcomes of different hospitals. However, our result of the subsample with a larger shifting effect is robust against the concern of the impaired childbirth outcome associated with the shifting of childbirth services.

This study has important implications for public health policy; in addition to the improved outcome of perinatal care, regionalization of high- and low-risk deliveries leads to better allocation of health-care resources and cost savings for the health-care system. In response to the escalation of health expenditures, health planners can not only maintain quality of care, but also better allocate resources and minimize costs by encouraging the use of less expensive healthcare facilities for low-risk deliveries whenever possible. Regionalized perinatal care ensures that high-risk deliveries that require more sophisticated equipment and care are referred to technologically advanced hospitals. The concentration of high-risk deliveries in a smaller number of advanced hospitals increases patient volume in neonatal intensive care units (NICUs), and leads to better outcomes for high-risk infants [19]. Hence, regionalized perinatal care has efficiently allocated expensive resources of advanced hospitals to high-risk infants who are most in need of help and has provided better quality of care for these babies.

In acknowledging governmental budget constraints and the need for efficient allocation of health-care resources to enhance the quality of childbirth care, we emphasize the importance of routine antenatal screening services and a well-established referral system for high-risk pregnancies. This study shows that it is possible to successfully shift low-risk deliveries from advanced hospitals to local community hospitals without impairing childbirth outcomes. The shift allows for a more efficient use of resources because low-risk deliveries seldom need high-technological medical facilities, such as NICUs. In addition, the shifting of childbirth services to local community hospitals would likely reduce patient travel and wait times and, thereby, increase the accessibility of care. However, public perception that technologically advanced hospitals provide a safer environment in which to deliver can discourage low-risk expectant mothers from using local community hospitals. Policy makers should therefore encourage pregnant women to seek childbirth services in local community hospitals in combination with providing antenatal screening services and appropriate referrals.

## Conclusion

Although it has not been documented conclusively whether or not advanced hospitals provide better care for normal birthweight deliveries than small maternity units [7-15], this study has demonstrated that childbirth outcomes were not influenced by the shift in maternity services to local community hospitals during the SARS epidemic in Taiwan. There was no significant change in neonatal and maternity mortality associated with the increased services in clinics and community hospitals, implying that local community hospitals provide similar quality of maternity care for low-risk births as advanced hospitals. Therefore, this study offers a potentially cost-efficient strategy for public health planners by providing evidence that can be used to encourage low-risk expectant mothers to seek childbirth services in local community hospitals. The provision of antenatal screening services and the implementation of an effective referral system for high-risk deliveries must also be in place.

## List of abbreviations

SARS: severe acute respiratory syndrome; IRB: Institutional Review Board; CI: confidence interval; NICU: neonatal intensive care unit; NHI: the National Health Insurance

## Competing interests

The authors declare that they have no competing interests.

## Authors' contributions

SYW planned and designed the study, collected and analyzed the data, interpreted the results, and led the writing. SHH contributed to the design and the analysis of the data, commented on the interpretation of the results, and completed the writing of the article. LKC collected the data and contributed to the interpretation of the results and writing of the article. All authors reviewed and approved the final draft of the manuscript.

## Pre-publication history

The pre-publication history for this paper can be accessed here:


